# A Rapid Detection Method of *Brucella* with Quantum Dots and Magnetic Beads Conjugated with Different Polyclonal Antibodies

**DOI:** 10.1186/s11671-017-1941-z

**Published:** 2017-03-09

**Authors:** Dandan Song, Xiaofeng Qu, Yushen Liu, Li Li, Dehui Yin, Juan Li, Kun Xu, Renguo Xie, Yue Zhai, Huiwen Zhang, Hao Bao, Chao Zhao, Juan Wang, Xiuling Song, Wenzhi Song

**Affiliations:** 1 Department of Health Laboratory, School of Public Health, Jilin University, 130021 Changchun, China; 2 State Key Laboratory of Inorganic Synthesis and Preparative Chemistry, College of Chemistry, Jilin University, 130000 Changchun, China; 3 China-Japan Union Hospital, Jilin University, 130000 Changchun, China; 4 School of Public Health, Xuzhou Medical University, 221000 Xuzhou, China

**Keywords:** Quantum dots, Immunomagnetic beads, IgY, IgG, Fluorescence

## Abstract

*Brucella spp.* are facultative intracellular bacteria that cause zoonotic disease of brucellosis worldwide. Traditional methods for detection of *Brucella spp*. take 48–72 h that does not meet the need of rapid detection. Herein, a new rapid detection method of *Brucella* was developed based on polyclonal antibody-conjugating quantum dots and antibody-modified magnetic beads. First, polyclonal antibodies IgG and IgY were prepared and then the antibody conjugated with quantum dots (QDs) and immunomagnetic beads (IMB), respectively, which were activated by *N*-(3-dimethylaminopropyl)-*N*’-ethylcar-bodiimide hydrochloride (EDC) and *N*-hydroxysuccinimide (NHS) to form probes. We used the IMB probe to separate the *Brucella* and labeled by the QD probe, and then detected the fluorescence intensity with a fluorescence spectrometer. The detection method takes 105 min with a limit of detection of 10^3^ CFU/mL and ranges from 10 to 10^5^ CFU/mL (*R* ^2^ = 0.9983), and it can be well used in real samples.

## Background

*Brucella* are gram-negative coccobacilli which can infect pigs, cattle, sheep, etc. According to its susceptible hosts, *Brucella* can be divided into *B. melitensis*, *B. suis*, *B. abortus*, *B. neotomae*, *B. ovis*, *B*. canis, *B. pinnipedialis*, *B. ceti* [1], *B. microti* [2], and *B. inopinata* [3, 4]. *Brucella*, especially *B. melitensis*, can cause abortion, stillbirth, and retention of placenta of dams and can cause a great loss of stock farming. It can also spread to humans through infected milk and contact with infected animals. As there is no efficient method to prevent pathophoresis, rapid detection of *Brucella* has become an issue attracting widespread attention.

The traditional detection methods of *Brucella* are the isolation identification method, the molecular biology method, and the immunological method, which each have different advantages and shortcomings. The isolation identification method can give qualitative and quantitative results, but it is time-consuming and requires strict experiment conditions and professional experimenters. Molecular biology method, such as PCR and RT-PCR, are popular because they are time-saving, labor-saving, and highly sensitive, but they require accurate and expensive instruments and professional operators. The immunological method is also widely used due to its high specificity. It achieves rapid detection while has also having cross-reactivity. Since the food sample matrix is complex and pre-treatment is time-consuming, the traditional detection technology cannot meet the requirements of food testing. Therefore, some new technologies are being used in food hygiene inspection.

The first of these is immunity magnetic bead (IMB) technology. Magnetic nanoparticles (MNPs) with super-paramagnetic effects show strong magnetism. These magnetic properties have been widely used in biochips, biosensors, biological detection, and in vitro diagnosis to simplify the detection method and improve the sensitivity and specificity of the detection method [5–9]. IMB technology is an immunological detection and separation technology based on the specific antigen-antibody reaction. First, the antibody coated with carrier magnetic beads specifically recognizes the antigen of the reaction medium to form an antigen-antibody complex. This composite directional movement occurs under the action of an external magnetic field to isolate the antigen. Immunomagnetic separation (IMS) based on IMB is a proven technique used for the enrichment of a range of bacterial genera from a variety of sample matrices [10–12].

The second of these detection technologies is the quantum dot (QD) fluorescence labeling technique. The QDs are coated by the antibody as probes to detect the antigen of the reaction medium, then use fluorescence spectroscopy to detect the antigen-antibody complex for quality and quantity. QDs are widely used in the field of luminescent biological probes due to their high photobleaching threshold, good chemical stability, broad excitation spectrum, narrow emission spectrum, strong light stability, long fluorescence lifetime, etc. [13, 14].

The last of the new detection technologies is yolk antibody (immunoglobulin Y, IgY) technology. By immunization of laying hens, the antibody is isolated from the eggs and applied to microorganism detection and disease treatment.

**Fig. 1:**
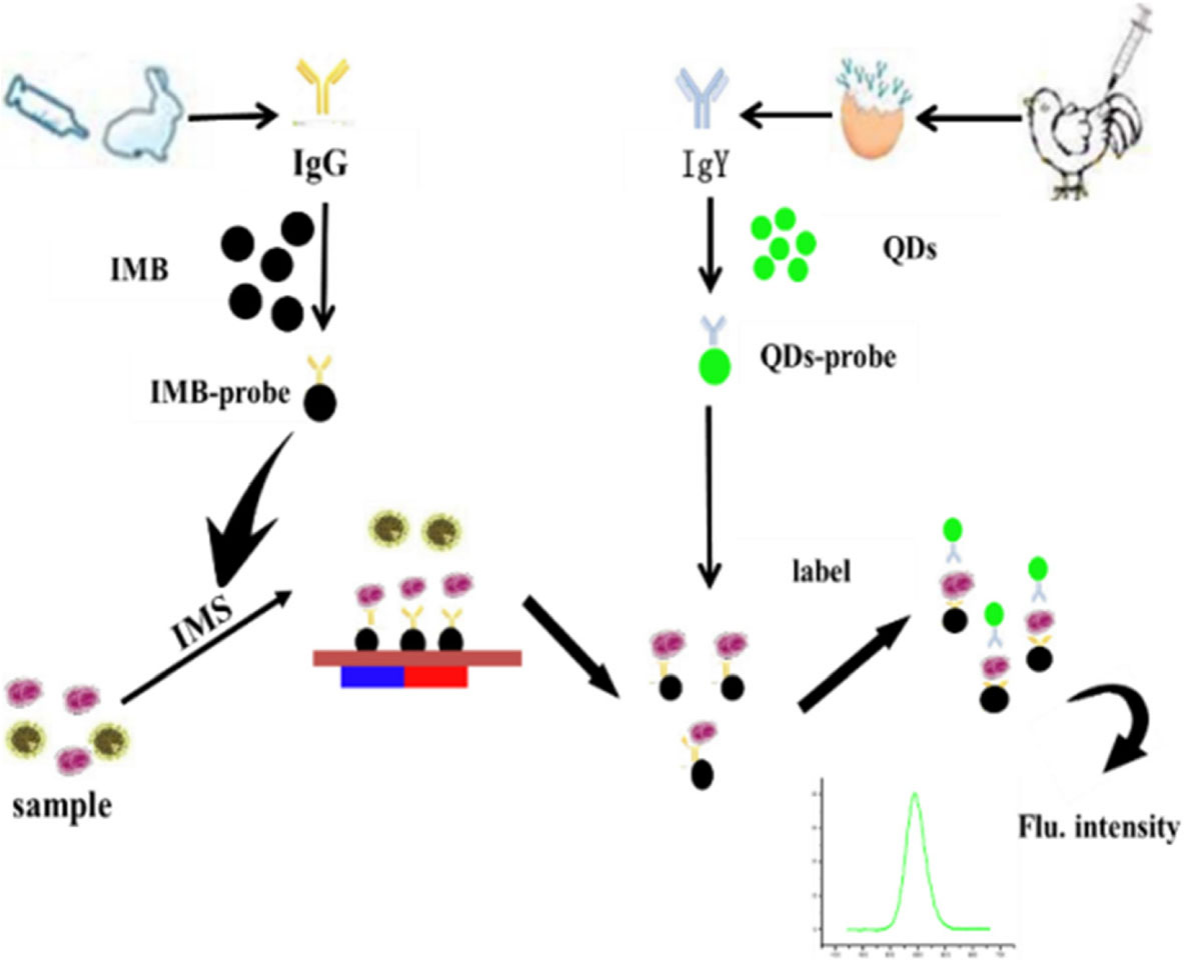
The technology road mapping. *Brucella* was separated by IMB probe, and then the QD probe was used as a fluorescent label probe to measure the fluorescence of the complex to determine whether *Brucella* is present

As the medium of food is complex and the amount of bacteria is too low to detect, we present a new method of *Brucella* detection with magnetic beads (MBs) and surface functionalized quantum dots (QDs), conjugated with different polyclonal antibodies to enrich pathogens and detect them rapidly and accurately [15] (Fig. 1).

## Results

### Evaluation of IgG and IgY

**Fig. 2:**
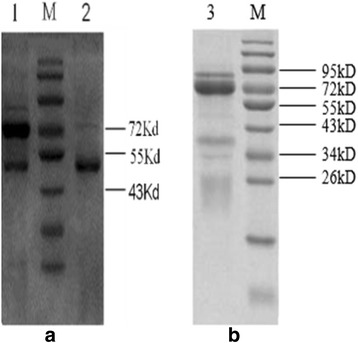
Assessment of the purity of IgG (**a**, *2*) and IgY (**b**, *3*). The rabbit serum (**a**, *1*) has more mingle strips than IgG, so the saturated ammonium sulfate method is efficient. *M* marker

The results of the purified IgG and IgY are as follows (Fig. 2). The molecular weight of IgG is 150 kD, the heavy chain is 53 kD, and the light chain is 22 kD. IgY is 180 kD, and it has two heavy chains that are between 60 and 70 kD, and two light chains that are between 22 and 30 kD (Fig. 2). The protein content of IgG is 17.454 mg/mL, and the protein count of IgY is 26.193 mg/mL.

The determination of the IgG (Table 1) and IgY (Table 2) titers and their specificity can be obtained by optimized indirect ELISA. The result was obtained and evaluated by the OD450 signal ratio of the positive to negative sample (P/N ratio). P/N ≥2.1 indicates positive, while P/N ≤2.1 designates negative. The titer of IgG is >1:256,000, and the titer of IgY is >1:2,048,000. This clearly demonstrates that the titer of the IgY can reach a stable level after a month of the first immunization and is much higher, more stable, and has better specificity than IgG.

**Table 1:** The titer of roughly purified rabbit antibody, IgG

Dilution	1:2000	1:4000	1:8000	1:16,000	1:32,000	1:64,000	1:128,000	1:256,000	Negative
Serum	2.757	2.528	2.626	2.257	1.739	1.170	0.700	0.387	0.065
IgG	2.4865	2.4682	2.4379	2.376	1.7194	1.368	0.8529	0.4622	0.070

**Table 2:** The titer of immunoglobulin of yolk, IgY

Dilution	1:8000	1:16,000	1:32,000	1:64,000	1:128,000	1:256,000	1:512,000	1:1,024,000	1:2,048,000
Negative	0.237	0.155	0.104	0.090	0.066	0.058	0.057	0.056	0.056
0609	0.242	0.127	0.082	0.076	0.065	0.061	0.059	0.060	0.060
0622	2.549	1.227	0.738	0.407	0.257	0.130	0.091	0.083	0.068
0723	2.530	2.537	2.591	2.256	2.097	1.452	0.817	0.549	0.362
0727	2.597	2.555	2.337	1.829	0.637	0.698	0.401	0.250	0.145

### Characterization of CdSe/CdS/ZnS QDs

**Fig. 3:**
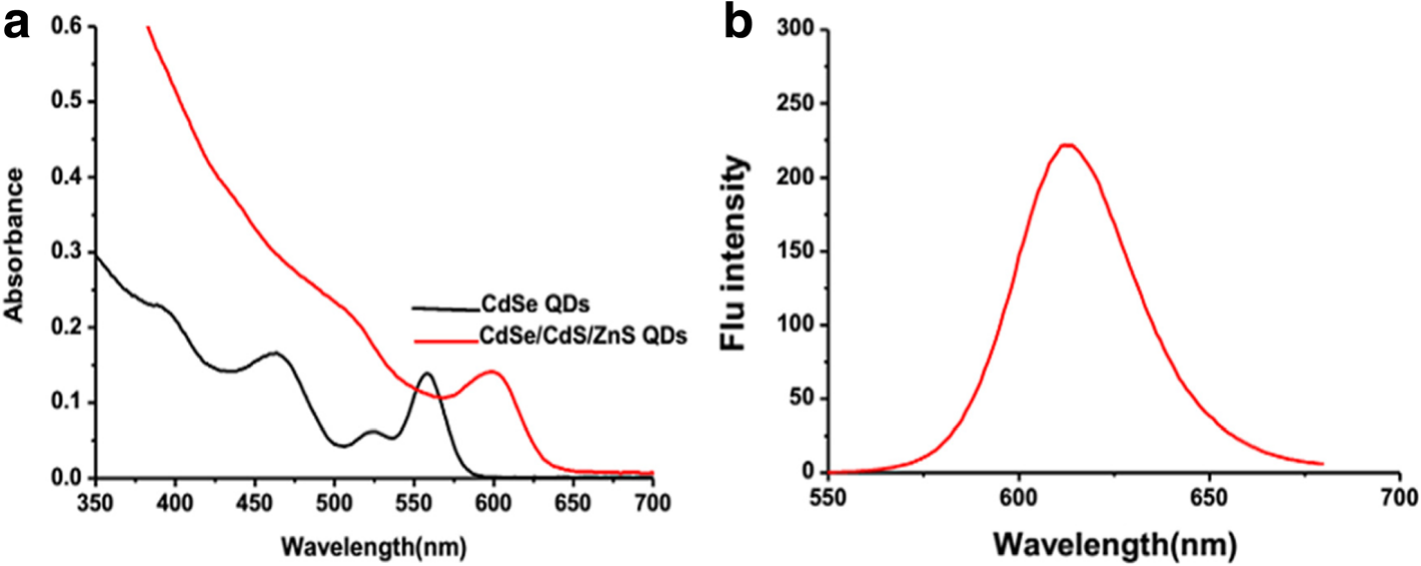
Characterization of CdSe/CdS/ZnS QDs. **a** Absorption spectra of CdSe QDs and the core-shell CdSe/CdS/ZnS QDs. **b** FL spectra of the water-soluble CdSe/CdS/ZnS QDs samples

**Fig. 4:**
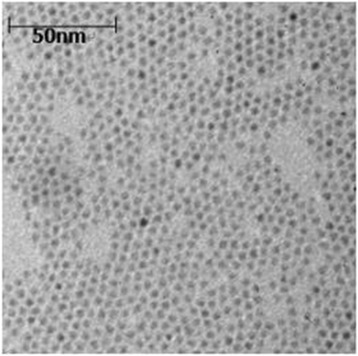
TEM images of CdSe/CdS/ZnS QDs

The QD solution was diluted 30 times with toluene. The UV-vis absorption peak occurred at 557 and 603 nm (Fig. 3a). The fluorescence intensity peak of the water-soluble QDs showed maximum intensity at 623 nm (Fig. 3b). High-resolution TEM was used to characterize QDs. Figure 4 shows the TEM image of the QDs, which indicates that the size of the QDs was about 4.6 nm. The UV-vis absorption and PL spectra of these QDs in the entire size regime are consistent with the nearly monodisperse size distribution of the nanocrystals confirmed by TEM.

### Preparation and Optimization of Probes

The fluorescence intensity of the complex was increased with the increase of the amount of IgY. When the flu intensity reached the highest level, the IgY is 262 μg, indicating that the most suitable amount of IgY is 262 μg.

**Fig. 5:**
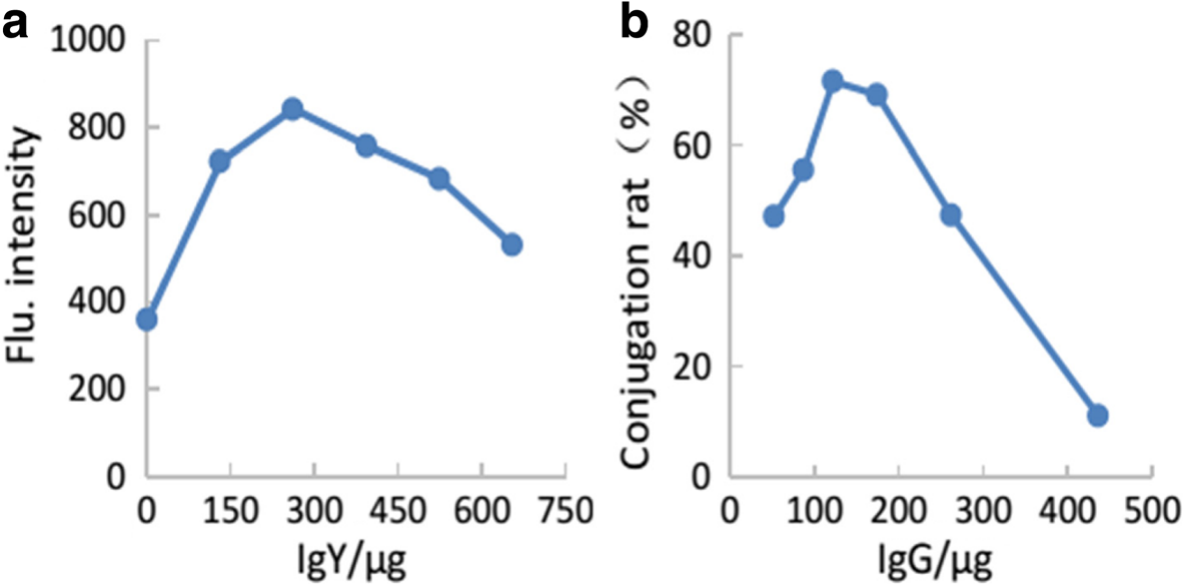
The optimization of bio-probes. **a** QD probe, the fluorescence intensity of the complex was increased with the increase of the amount of IgY. The flu intensity reached the highest level and then stepped down. **b** MB probe, A280 nm of the IgG solution was determined before and after conjugation, and the amount of conjugated IgG of IMB was calculated

The IMB was activated by NHS-EDC to react with the amino of proteins, so when a certain amount of IgG was added, it could be conjugated on the surface of the IMB. As shown in Fig. 5b, as the amount of IgG was increased, the conjugation rate gradually increased, and when IgG reached 122.178 μg, the conjugation rate reached the highest level which can be determined as the optimal amount of IgG.

### Establishment of Detection Method

**Fig. 6:**
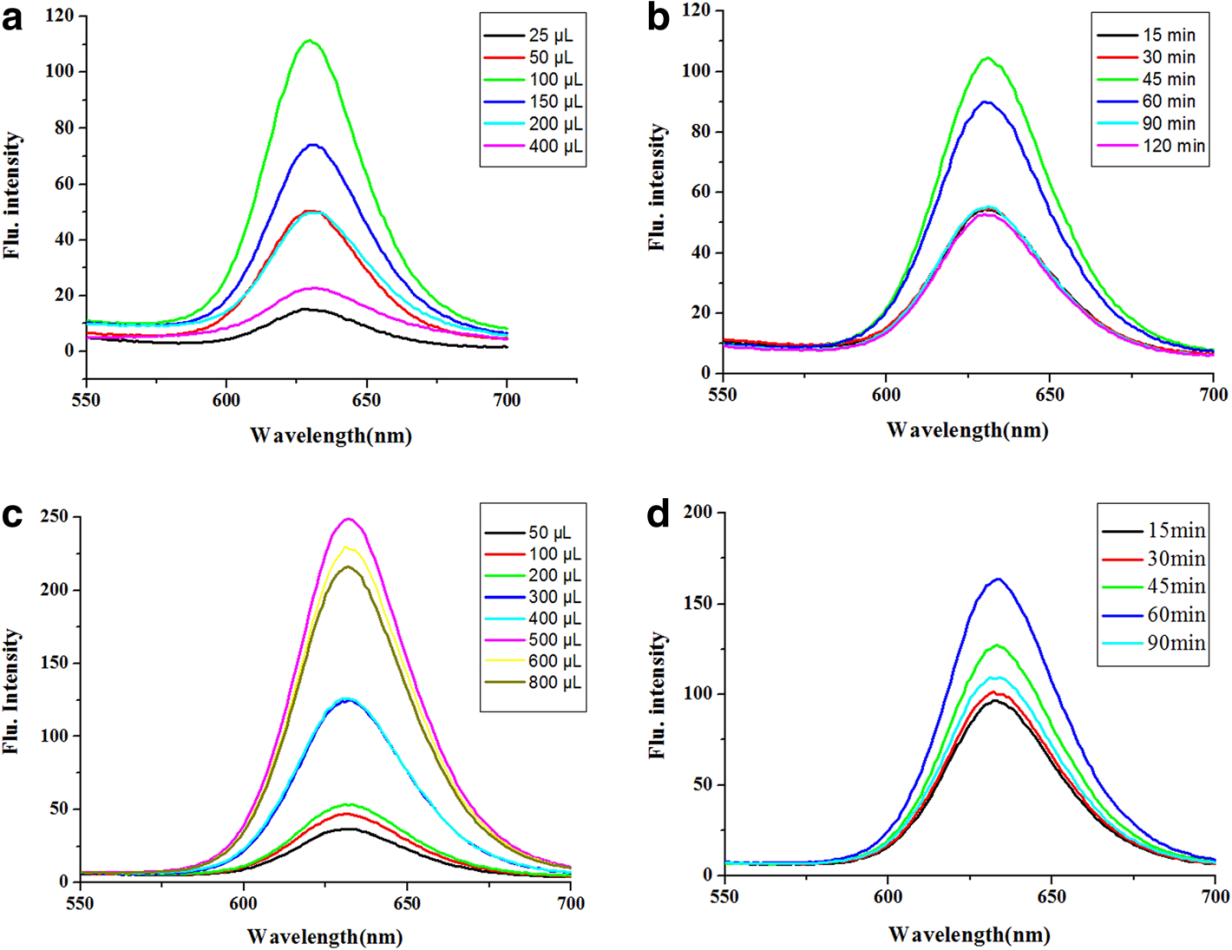
Results of the optimization of the detection conditions. **a** The optimization of the amount of IMB probe. **b** The optimization of the IMS. **c** The optimization of the amount of QD probe. **d** The optimization of the mark time

To establish a method with high sensitivity and low detection limit for *B. melitensis* 16M, it is essential to optimize the main parameters involved in the process. As shown in Fig. 6a, the fluorescence intensity increased as the amount of the IMB probe increased, and the flu intensity was reduced following the IMB probe increase above 100 μL, indicating that the optimal IMB probe amount is 100 μL. This may be due to the partial aggregation of IMB, which reduces the florescence escape efficiency. Similarly, Fig. 6b indicates that the fluorescence intensity of the complex reached the maximal value at the reaction time of 45 min, which was then used as the optimal time for IMS. Another important factor that affects the flu intensity of the conjugation product is the amount of the QD probe. The optimal amount is 500 μL (Fig. 6c). Finally, the optimal mark time is 60 min (Fig. 6d).

### Sensitivity and Specificity of the Method

**Fig. 7:**
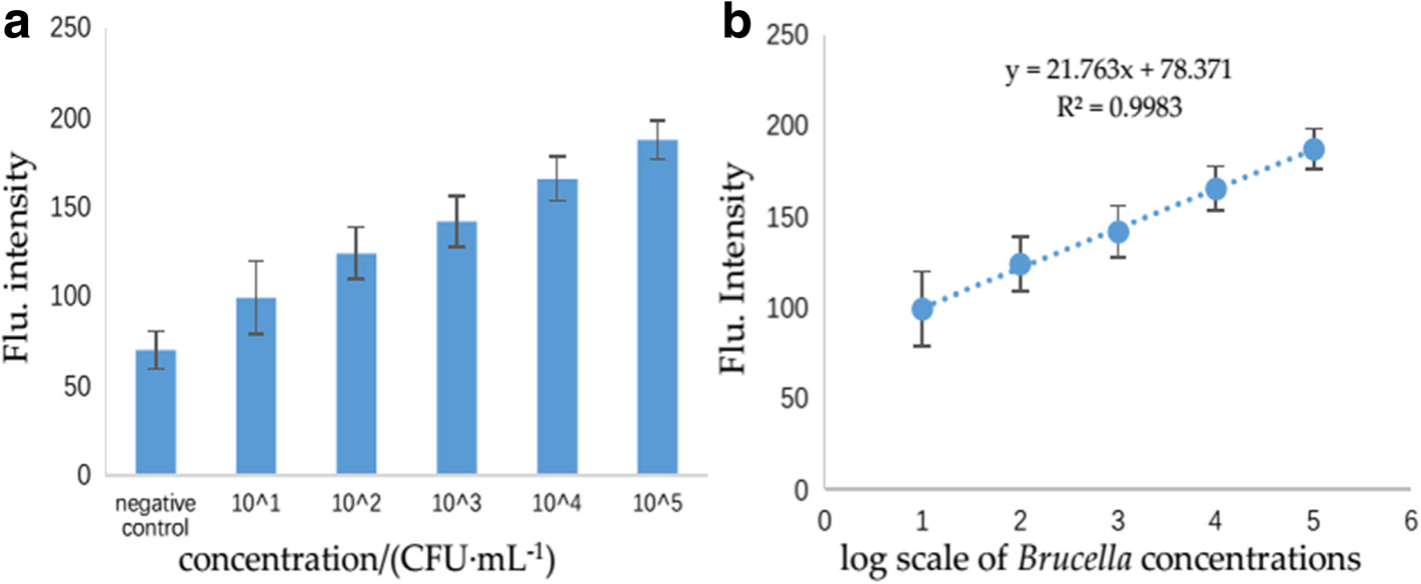
**a** Results of sensitivity of the detection method of *Brucella*. **b** P/N ≥2.1 indicates positive. **c** the LOD is 10^3^ CFU/mL

Based on the optimum conditions of the detection method, 100-μL IMB probe was added into different concentrations of *B. melitensis* 16M for 45 min. After separation and washing by PBST, 500 μL QD probe was added in the reaction system. After 1 h incubation, the complex was detected and was examined by a fluorescence spectrometer. The determination of LOD was calculated by the flu intensity ratio of the positive to negative sample (P/N ratio). P/N ≥2.1 indicates positive, on the contrary is negative. Thus, 10^3^ CFU/mL was defined as the LOD of the detection method (Fig. 7a) [15, 16]. We added 10^3^ CFU/mL to the detection system and the flu intensity is 142.714. According to the standard curve (*y* = 21.763× + 78.371, R^2^=0.9983), we can calculate the *x* = 2.957. So the recovery rate = 10^2.957^/10^3^ = 90.57% (Fig. 7b).

**Fig. 8:**
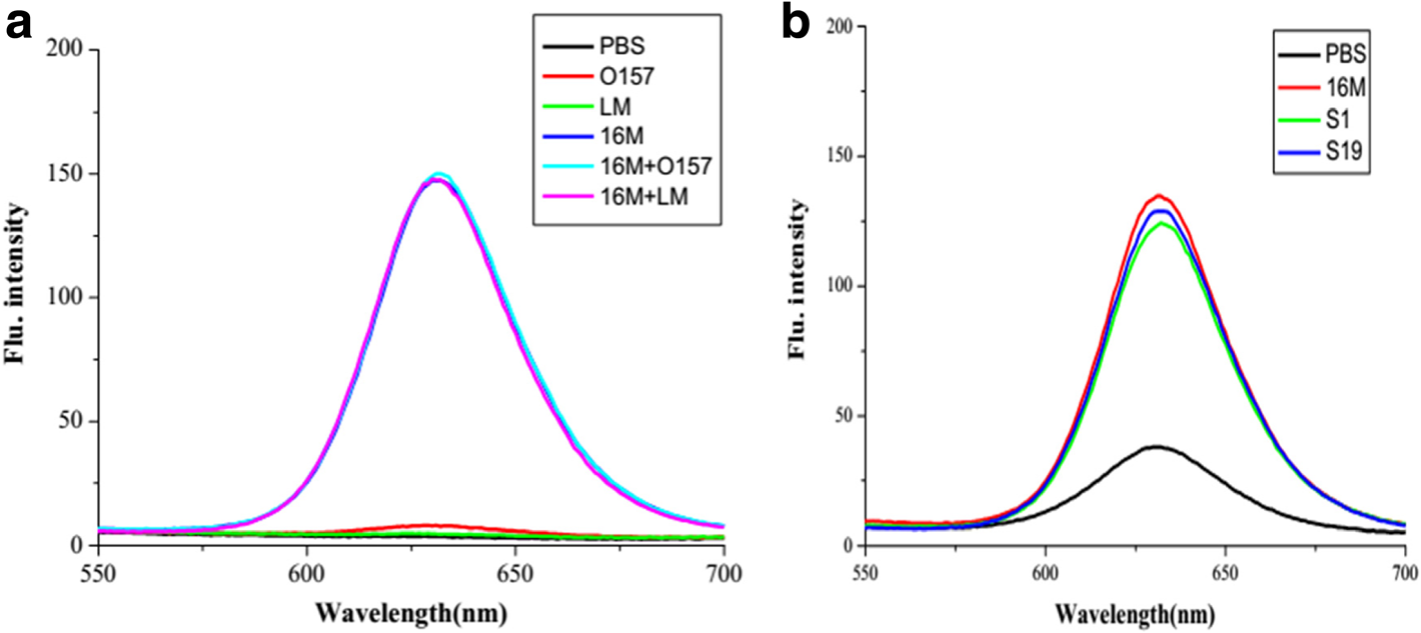
The specificity of test of the detection method. **a** The specificity inter-species. **b** The specificity intraspecies, the flu intensity is similar among those intraspecies

The specificity of this detection method, evaluated by the comparison of the results of *Liseria monocytogenes*, *E. coli* O157, and *B. melitensis* 16M is shown in Fig. 8a. It shows that the fluorescence intensity of LM and O157 was not significantly different from the negative control, and the fluorescence intensity of the mixtures of *B. melitensis* 16M and LM, and *B. melitensis* 16M and O157 were not fundamentally different from *B. melitensis* 16M alone. Figure 8b also illustrates the detection of S1 and S19 and shows that the fluorescence intensity of S1 and S19 were not different from *B. melitensis* 16M alone, indicating that the method cannot recognize the intraspecies well. This allows us to draw the conclusion that the method can recognize the target bacteria with high specificity.

### Detection in Real Samples

**Fig. 9:**
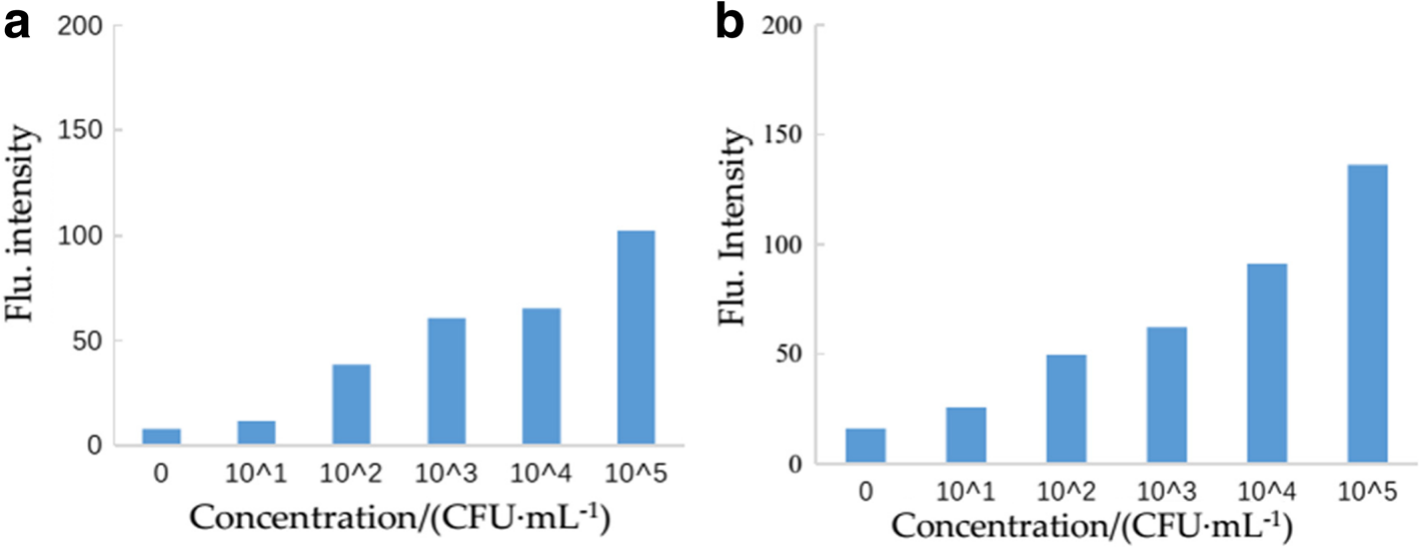
The result of inoculated sample detection. **a** Milk. **b** Lamb

To further investigate the potential practical applications of the method, it was applied to detect certain concentration of *B. melitensis* 16M in milk and lamb-steep liquor. As Fig. 9 shows, the LOD of *B. melitensis* 16M is 10^2^ CFU/mL, demonstrating that this detection method can be used for *Brucella* detection in real samples with satisfactory results.

## Discussion

The fluorescence intensity of QDs is stable, and when conjugated with IgY, it may steadily rise then to reach a highly stable level. While Fig. 6a illustrates the fluorescence intensity of the QD probe’s optimization, the reason for its increase and subsequent decrease needs to be intensively studied.

The total detection process lasts 105 min which is time-saving compared with those without pre-enrichment, and they need enhancing culture to detect bacteria which is more time-consuming. When the detection method is used in real samples, such as milk, it can be seen that the fluorescence intensity is lower compared with the ideal samples and the LOD can be defined as 10^2^ CFU/mL. The reason for this may be the matrix in milk and lamb is more complex. And there may be competition in the epitope of *B. melitensis* 16M. As the epitope is conjugated with other proteins, the amount of QD probe is reduced and the flu intensity is decreased.

Previously, Liandris et al. established a detection method of *mycobacteria* with functionalized QDs coupled with IMS [17]. The principle is based on the separation of bacteria with magnetic beads and QDs coupled with genus-specific polyclonal antibody and monoclonal antibody for heparin-binding hemagglutinin. The detection limit was 10^4^ CFU/mL. Yang et al. also built a method with functionalized QDs and IMB, and they coupled it with phage various antibodies and display-derived peptides, and the LOD was determined to be 10^3^ CFU/mL and the time of the test was more than 2 h [15]. Wu et al. established a simple and rapid method using electrochemical impedance spectroscopy (EIS) to detect *Brucella*, and its LOD is 10^4^ CFU/mL [18] (Table 3). In this assay, we introduced IgY as the specificity antibody. Known for its better thermal and pH stability, high sensitivity and specificity, low cost, high yield, ease of purification, etc. than other antibodies, it has become more and more popular. Also it is obvious to see that the detection method in this assay is more time-saving and with higher sensitivity with the limit 10^3^ CFU/mL and range from 10^1^ to 10^5^ CFU/mL (*R* ^2^ = 0.9983).

**Table 3:** A comparison of the performance between different assays

	LOD (CFU/mL)	Time (h)	Linear
Liandris et al. [17]	10^4^	–	–
Yang et al. [15]	10^3^	2	–
Wu et al. [18]	10^4^	–	–
Our study	10^2^	1.5	10^2^–10^5^

## Conclusions

All in all, the detection method of *B. melitensis* 16M with an IMS of 45 min, a label of 60 min, and an entire duration of 105 min or more, is time-saving compared to traditional methods and can be used with satisfactory results in milk and lamb-steep liquor with 10^2^ CFU/mL. With the stable specificities of the QD probe, we can further establish a method to detect two or three bacteria in the same system, which is important for public health.

## Methods

### Source of Strain

*B. melitensis* 16M (virulent strain), *B. suis*, strain S1 (S1). and *B. abortus*, strain 19 (S19) were gathered from the *Brucellosis* Prevention and Control Base, Chinese Centers for Disease Control and Prevention, Baicheng City, Jilin Province, China. The hens were bought from the HaoTai Laboratory Animal Breeding Co., Ltd, Shandong. The New Zealand white rabbit was bought from the Experimental Animal Center of Jilin University.

### Animal Immunization

The animal procedures were all carried out according to the Animal Scientific Procedures Act (1986) and the guidelines of the Agri-Food and Biosciences Institute (AFBI) Ethics Committee. Polyclonal antibodies were produced by injecting rabbits and hens with formaldehyde-inactivated *B. melitensis* Freund’s complete adjuvant vaccine and boosters using Freund’s incomplete adjuvant vaccine. The antibodies’ titer of rabbit and hen serum was monitored by indirect enzyme-linked immunosorbent assay (iELISA) regularly. When the titer of serum antibodies reached a highly stable level, the inactivated bacteria were used to finalize immunity.

### Antibodies Production

The two rabbits were executed by drawing-out all the blood in their hearts 10 days after final immunization. All serum was collected by centrifugation of blood for 10 min at 3000 rpm. Then the serum was purified by 50 and 33% saturated ammonium sulfate, and the polyclonal antibody immunoglobulin G (IgG) was obtained. The hen eggs were collected and IgY was extracted by precipitation with polyethylene glycol (PEG) [19].

### Evaluation of IgG and IgY

The purified IgG and IgY with SDS-PAGE were identified according to the manufacturer’s instructions with a 5% stacking gel and a 12% separating gel. The protein content was determined by BCA kit. Antibody titers were monitored by optimized indirect ELISA. Microtiter plates were coated with inactivated *B. melitensis* 16M (virulent strain) and incubated overnight at 4 °C. Then washed three times with PBST buffer solution (0.01 M PBS, pH 7.4, containing 0.05% Tween-20). After blocked by 5% skim milk powder (*g*/*v*, PBST buffer solution) for 1 h at 37 °C and washed by PBST, double diluted IgG or IgY was added to the plates for 1 h at 37 °C. After a washing step, a 1:10,000 diluted HRP-goat anti-rabbit IgG secondary antibody and HRP-goat anti-chicken IgG secondary antibody was added accordingly and incubated at 37 °C for 0.5 h. After the washing step, 100 μL of TMB substrate solution was added to each well and held for 20 min at room temperature in a dark place. Finally, 50 μL of H_2_SO_4_ (2M) was added to each well. ODs at 450 nm were measured by an ELISA plate reader (BioTek, USA).

### Quantum Dots Preparation and Characterization

CdSe quantum dot suspension was prepared according to the literature with a slight modification [20]. CdO (0.20 mmol) and OA (1 mmol) in ODE (1.5 mL in total) were first prepared as the Cadmium precursor. The Se-TOP was prepared by Se powder (1 mmol) in 5 mL TOP and 5 mL ODE by nitrogen bubbling for 10 min. OLA (1 mL), Cadmium precursor (2 mL), and ODE (2 mL) were loaded into a 50-mL three-neck flask. After stirring and vacuum pumping for 20 min at 80 C, the solution was heated to 240 C with nitrogen bubbling. Se-TOP (1 mL) was quickly injected into the solution at 240 C for growth of the CdSe QDs. Needle-tip samples were taken by the syringe and were dissolved in toluene for UV-vis and PL measurements.

Good fluorescence performance of core-shell CdSe/ZnS QDs was due to the surface passivation by ZnS [21, 22]. CdSe solution (3 mL), ODE (1 mL), and OLA (1 mL) were fixed to the three-neck flask. The Cd(OA)2 solution (0.2 mmol, Cd: OA = 1:4) was injected at 130 C. After 20 min, aliquots of S-ODE solution (0.2 mmol) were injected slowly and reacted for 30 min at 240 C. For the ZnS shell, equal molar ratios of Zn(OA)2 (0.2 mmol, Zn: OA = 1:6) and S-ODE were used as precursors. The Zn(OA)2 solution was injected at 130 C and showed growth at 220 C. The S-ODE solution was injected at 150 C and showed growth at 240 C. The same process was repeated twice. After the reaction was complete, the reaction solution was centrifuged to extract the unreacted materials and byproducts. The resulting solution was dispersed in chloroform.

In order to obtain the solution of water-soluble quantum dots, aliquot chloroform with CdSe/CdS/ZnS quantum dots was used. 1 mL of CdSe/CdS/ZnS chloroform solution, 1 mL of mercaptopropionic acid, and 2 mL of deionized water were added to the bottle, stirring for 2 h. The quantum dots were transferred from the initial chloroform solution to the aqueous solution, and, after centrifuging three times, the quantum dots scattered in the deionized water.

### Preparation and Optimization of QD Probe

The IgY was covalently bonded to QDs by coupling agents *N*-(3-dimethylaminopropyl)-*N*’- ethylcar-bodiimide hydrochloride (EDC, Sigma) and *N*-hydroxysuccinimide (NHS, Sigma) in MEST [23]. The 10 μL QDs were mixed with 200 μL NHS (10 mg/mL) and 200 μL EDC (10 mg/mL). The mixture was placed in a vertical mixer (Qilinerier, China) and was shocked for 30 min at room temperature. Then, the solution was centrifuged at 12,000 rpm for 5 min and washed three times with phosphate-buffered saline with Tween® (PBST), followed by adding a different amount of IgY and incubating for another 2 h in the vertical mixer. It was then washed three more times with PBST blocked by 500 μL 1% BSA (*g*/*v*, PBS buffer solution) for 1 h. After washing with PBS, the complex can be re-dispersed by 200 μL PBS and stored at 4 C. This provides the optimum amount of IgY by comparing the fluorescence of the complex elevated by a fluorescence spectrometer at 632 nm.

### Preparation and Optimization of IMB Probe

Preparation of the IMB probe followed the manufacturer’s instructions. The concrete steps were as follows: 100 μL IMB (BeaverBeads™ MagCOOH, 2 μm) was washed by MEST (100 mM MES, pH 5.0, 0.05% Tween® 20) and was separated by magnetic device three times before adding 200 μL EDC (10 mg/mL, MEST as dispersant) and 200 μL NHS (10 mg/mL, MEST as dispersant) to the IMB shocked by the vertical mixer for 30 min at 25 C. This was washed three times with PBST, and different amounts of IgG were added and shocked by the vertical mixer for 2 h. The solution was then separated by a magnetic device, with the supernatant saved as a standby, then washed three times with PBST, added to 500 μL 1% BSA (*g*/*v*, PBS buffer solution), and shocked for 1 h. After blocking and washing, the resultant IMB-IgG bio-conjugations were then re-dispersed in 400 μL PBS and stored at 4 C standby. We measured the absorbance at A280 nm by the ultraviolet spectrophotometer (Persee, China) to confirm the optimal amount of IgG [24].

### Establishment of Detection Method

*B. melitensis* 16M was separated by an IMB probe, and then the QD probe was used as a fluorescent label probe to measure the fluorescence of the complex to determine whether *Brucella* is present. To establish the detection method, we can optimize the step of the method as the amount of the IMB probe, time of IMS, the amount of QD probe, the label time, etc. [15, 25].

The determination of the limit of detection (LOD) can be operated as follows [15]: we prepared a series of tenfold dilutions, ranging from 10 to 10^5^ CFU/mL, the LOD was defined as the lowest concentration levels of *B. melitensis* 16M, which were statistically different from the negative control [15]. The specificity of this detection method was evaluated by the comparison of the sensing results of *L. monocytogenes* (ATCC 19115), *E. coli* O157 (ATCC 35150), and *B. melitensis* 16M. Intraspecies specificity can be evaluated by detection of *B. suis*, strain S1(S1), and *B. abortus*, strain 19 (S19)20, 21.

### Model Sample Detection

The most certain method was used to detect the samples of aseptic milk (Yili) and lamb-steeped liquor (Haoyue) [26]. A series of different concentrations of 16M were artificially inoculated into the pasteurized milk and lamb-steeped liquor (the lamb liquid lapping compound soaked by saline for 24 h) and then detected the 16M with this method as described above.

## Abbreviations

IMB: Immunomagnetic beads
IMS: Immunomagnetic separation
QD: Quantum dots
